# The Effect of Silver and Copper Nanoparticles on the Wheat—*Pseudocercosporella herpotrichoides* Pathosystem

**DOI:** 10.1186/s11671-017-2028-6

**Published:** 2017-04-04

**Authors:** V. N. Belava, O. O. Panyuta, G. M. Yakovleva, Y. M. Pysmenna, M. V. Volkogon

**Affiliations:** Educational and Scientific Centre, Institute of Biology and Medicine, Taras Shevchenko National University of Kyiv, 64, Vladymyrska Str., Kyiv, 01601 Ukraine

**Keywords:** Biogenic metal nanoparticles, Wheat seedlings, *Pseudocercosporella herpotrichoides*, Eyespot causal agent, TBARS

## Abstract

The paper covers the study of the effects of silver (Ag) and copper (Cu) nanoparticles on wheat—*Pseudocercosporella herpotrichoides* pathosystem in general and, separately, on their interaction both with the plant and with the pathogen. Plants, treated with nonionic colloidal solutions of biogenic metal nanoparticles of Ag and Cu, have taken seed treatment as stress and have demonstrated the same changes in the dynamic patterns of thiobarbituric acid reactive substances (TBARS) content as a seedling infection or in its combination with a nanoparticle treatment. The wheat variety, which is sensitive to pathogen action, has showed a substantial (100%) increase in the TBARS contents, while the other varieties has shown lesser (40%) changes in the TBARS content as compared to the control. Besides, both silver and copper nanoparticles have not affected the growth and development of *P. herpotrichoides*, thus suggesting that the effect of nanoparticles is determined by the plant’s responses to the pathogen rather than the phytotoxic action of the copper or silver nanoparticles, at least during the initial stages of the pathological process.

## Background

Nanoparticles are of great interest and are widely used around the world nowadays. Their presence in croplands, coming either from other nanotechnology waste products (sewage, gas, etc.) or from their deliberate use as an alternative to traditional agricultural xenobiotics, used to improve crop production profoundly alarms the scientific community. Different studies have shown that metal and metal oxide nanoparticles influence a plant’s growth and development, crop yield, and quality [16]. Nanoparticles may affect plants on biochemical, physiological, and molecular levels through changes in mineral nutrition and photosynthesis. They cause oxidative stress and induce genotoxicity in crops. The enzyme activity of pro- and antioxidant systems is also highly dependent upon the concentration of nanoparticles [16].

Thus, the study of the cytotoxic, genotoxic, and biochemical impact of ZnO nanoparticles on *Vicia faba* and *Nicotiana tabacum* plants has revealed an increased intracellular synthesis of reactive oxygen species (ROS), a higher lipid peroxidation (LPO), and a higher antioxidant enzyme system activity. Research focused on the meristem roots of *Allium cepa* has shown a loss of membrane integrity, increased chromosome aberrations, micronucleus formation, breaks in the DNA strands and cell-cycle arrest at the G2/M checkpoint [5].

Vecerova et al. have showed that cadmium nanoparticles (CdO) significantly affected the total content of primary plant metabolites (amino acids and sugars) without a substantial impact on the total content of the secondary metabolites (phenolic compounds, Krebs cycle acids, and fatty acids) but changed the content of the saturated and unsaturated fatty acids in the roots and leaves of treated plants [22].

Another study has showed that treatment of *Pisum sativum* seedlings with argentum (Ag_2_O) nanoparticles significantly reduced their growth, the content of photosynthetic pigments, and chlorophyll fluorescence. The levels of oxide stress markers (SOR, H_2_O_2_, and MDA) have increased significantly under the action of Ag_2_O nanoparticles, followed by the stimulation of superoxide dismutase and ascorbate peroxidase activity, and the reduction of the total amount of ascorbate and glutathione in the tissues of the leaves and roots of the plants studied. According to Tripathi, the observed negative changes are associated with oxide stress and elevated levels of argentum in plant tissues [21].

The treatment of *Hydrilla verticillata* culture with titanium (TiO_2_) nanoparticles has reduced the enzyme activity of the plant’s antioxidant defense mechanisms. Moreover, different concentrations of TiO_2_ nanoparticles have resulted in a decreased GSH/GSSG ratio, indicating a high-GSH-dependent metabolic activity, which protects plants against the damage caused by the ROS generated as a result of the plants’ exposure to TiO_2_ nanoparticles [13].

Based on a study of the activity of antioxidant enzymes (superoxide dismutase and catalase) and the changes in thiobarbituric acid reactive substances (TBARS), the contents in soybean plants that were treated with a colloidal solution of biogenic metal nanoparticles (Ag, Cu, Fe, Zn, Mn), an exposure to even a small concentration of nanoparticles during specific growth stage, are perceived by the plants as a low-level stress factor, which, according to the principle of hormesis, promotes an appropriate adaptive response reaction by the plants [19].

Known antibacterial properties of metal nanoparticles are also of great interest to researchers [15]. According to the literature data, metal nanoparticles have a wide spectrum of biocidal properties against pathogens (coliform bacteria (*Escherichia coli*), streptococci, staphylococci, blue pus bacillus (*Pseudomonas auruginosa*), viruses, and molds). In particular, a significant fungicidal effect of silver nanoparticles was observed against *Penicillium citrinum*, *Aspergillus niger*, *Aspergillus flavus*, *Aspergillus fumigatus*, *Aspergillus terreus*, *Fusarium moniliforme*, *Trichophyton mentagrophytes*, and *Candida albicans* [17]. However, very little is known about the impact of metal nanoparticles on pathogenic fungi, while the importance of the impact of nanoparticles on complex biological systems remains high. One of the studies of the plant-microorganism system (on the model of red clover and its symbiotic microorganisms) has showed that nanoparticles affect plants and symbiotic microorganisms, significantly reducing the plants’ biomass, root colonization by symbionts, nodulation activity, and the flowering ability of plants [12].

However, it is believed that most nanoparticles can have both positive and negative effects on agricultural crops, depending on the crop and its growth stage, tillage, nutrition, applied nanoparticles, etc. [16]. In addition, the active influence of nanoparticles is expressed not only thorough changes in the metabolism of plants or plant symbiont systems. Nanoparticles might have a complex effect on all organisms, which stimulated our interest in studying the impact of biogenic metal nanoparticles on plant-pathogenic fungal systems. As the introduction of intensive technologies expands around the world and leads to the disruption of the ecological balance in croplands and the spread of crop diseases which previously had no special significance, this study becomes more urgent.

## Methods

To understand the nature of the impact of nanoparticles on the relationships within a plant-pathogen fungal system, two separate experiments were conducted. The first one was focused on the impact of biogenic metal (Ag and Cu) nanoparticles on the plant growth and development on and without an infection background (sand culture). Another experiment was aimed at revealing the effect of Ag and Cu nanoparticles directly on a pathogenic factor (the speed of fungus mycelium growth on an agar medium).

The plant experiment was performed with three varieties of winter wheat plants (*Triticum aestivum* L.) which possess different susceptibility to wheat eyespot pathogen agent (*Pseudocercosporella herpotrichoides*):

- Myronivska 808 (a Ukrainian selection)—a variety, sensitive to pathogen action;
- Roazon (a French selection)—a relatively resistant variety, which has the highest level of resistance in the world; and
- Renan—a hybrid variety, which resulted from breeding four varieties, including the abovementioned Myronivska 808 and Roazon.

The direct contact between nanoparticles and seed/plant roots is an essential requirement for a phytotoxicity study in plant model experiments [6]. Taking that into account, a pre-sowing wheat seed treatment with colloidal solutions of nanoparticles was performed using biogenic nanoparticles of silver and copper elements with well-known fungicidal properties.

The biogenic metal nanoparticles were obtained through the dispersing of corresponding metal granules with pulses of electrical current (amplitude 100–2000 A) in water (the Department of Material Technology and Material Science (the Faculty of Design and Engineering, the National University of Life and Environmental Sciences of Ukraine)) [10]. The maximum size of the nanoparticles has not exceeded 100 nm. The content of the nanoparticles in colloidal solutions was as follows: Ag + Ag_2_O, 1.5 mg/l; Cu, 0.75 mg/l.

Sterilized seeds were soaked in a working solution (the recommended concentration: 1 part of colloidal solution per 100 parts of water) for 4 h (distilled water in the control), washed with distilled water, and placed in an incubator (25 °C) for 24 h. The plants were grown on sand in chemically neutral containers under controlled laboratory conditions (16-h photoperiod, light intensity 15 000 lx, air temperature 25/20 °C (day/night), air humidity 60%); 25–35 ml of the Hoagland-Arnon nutrient solution was added to each container. The moisture level of the substrate was maintained at a constant (70%) level using an additional nutrient solution.

As the model pathogen, we used the high virulent 543 7/1 strain of wheat eyespot agent (*Pseudocercosporella herpotrichoides* (Fron) Deighton) (another name—*Oculimacula yallundae* (Wallwork and Spooner) Crous and W. Gams) [7], which is known for blocking fiber bundles and straw necrosis, reducing crop productivity and a massive amount of wheat lodging. Seven-day-old wheat seedlings were infected with a suspension of pathogenic fungi conidia (titer 5–7 × 10^4^ CFU/cm^3^) [2].

It is known that plant cells respond to stressors of different nature by giving an oxidative burst, which is associated with the rapid accumulation of ROS. Lipid peroxidation is one of the markers used to estimate the amount of damage caused by the oxidative burst, which is accompanied by breaching the structural and functional integrity of cell membranes. Using an accumulation of lipid peroxidation products as a marker, it is possible to assess the level of plant stress and its resistance to a given stressor [1]. In our experiments, the intensity of lipid peroxidation was evaluated daily for 7 days after infection, using 2-thiobarbituric acid (TBA) reactions, and was expressed as the content of thiobarbituric acid reactive substances in plants (TBARS, the main component of malonic dyaldehid (MDA)) [23]. Light absorption was recorded at λ 532 nm.

The impact of silver and copper nanoparticles on the growth rate of *P. herpotrichoides* mycelium was determined by surface fungal cultivation on an agar medium with a solution called biogenic metal nanoparticles.

The parent mycelium of *P. herpotrichoides* was grown in test tubes and was stored on a slant potato dextrose agar (PDA) medium, which conforms to the culture media used for cultivation of *P. herpotrichoides* [3].

Solutions of metal nanoparticles, silver or copper, were added to the culture medium at a concentration of 1:100 (1 part of nanoparticles solution per 100 parts of PDA medium or 1.5 mg/l and 0.75 mg/l, respectively). A control PDA medium was used without the metal nanoparticles solution. Preparing and sterilizing culture medium were performed according to conventional methods [11].

A medium of 25 ml was poured in each Petri dish. A 14-day culture of *P. herpotrichoides*, grown on PDA in Petri dishes, was used for the experiments. Agar discs with fungus mycelium (*d* = 10 mm), cut with a sterile drill from the edge of actively growing colonies, were transferred to the center of the Petri dish (*d* = 90 mm). The inoculation was performed under sterile conditions. The cultivation of *P. herpotrichoides* mycelium was conducted at 25 ± 1 °C for 14 days [4].

The radii of the colonies were measured in four mutually perpendicular directions every day from the first day after the plates were inoculated until the dish was completely overgrown.

The average radial growth rate of the colonies (*V*_*r*_) was calculated using the following formula:

$$ {V}_r=\frac{R_1-{R}_0}{n},\ \mathrm{mm}/\mathrm{day}, $$

where *R*_1_ is colony radius in the late phase of linear growth (limited by the size of the Petri dish), in mm; *R*_0_ is the initial colony radius at the early stage of linear growth, in mm; and *n* is the duration of the linear growth, in days.

Threefold biological and ninefold analytical repetitions of the experiments were performed. The results were statistically processed using conventional methods. All samples were subject to the principle of normal distribution. The data were considered significant at *P* < 0.05.

## Results and Discussion

**Fig. 1:**
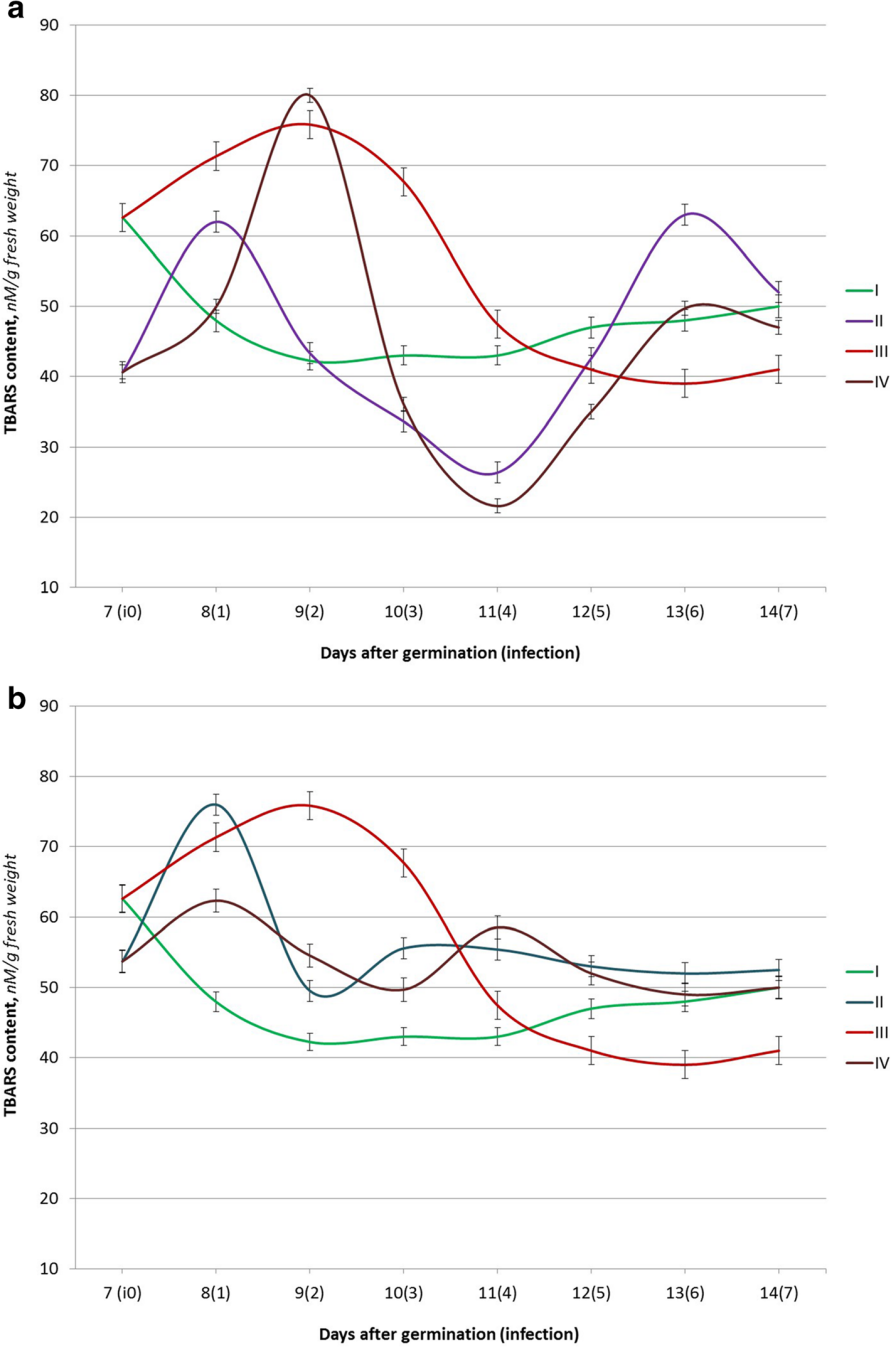
The TBARS content in seedlings of winter wheat (the Myronivska 808 variety) under the action of Ag (**a**) or Cu (**b**) nanoparticle solutions and *P. herpotrichoides* infection: *I* control plants (w/o infection); *II* seed treatments with Ag (**a**) or Cu (**b**) nanoparticle solution; *III P. herpotrichoides* infection; *IV P. herpotrichoides* infection + treatment with Ag (**a**) or Cu (**b**) nanoparticle solutions

**Fig. 2:**
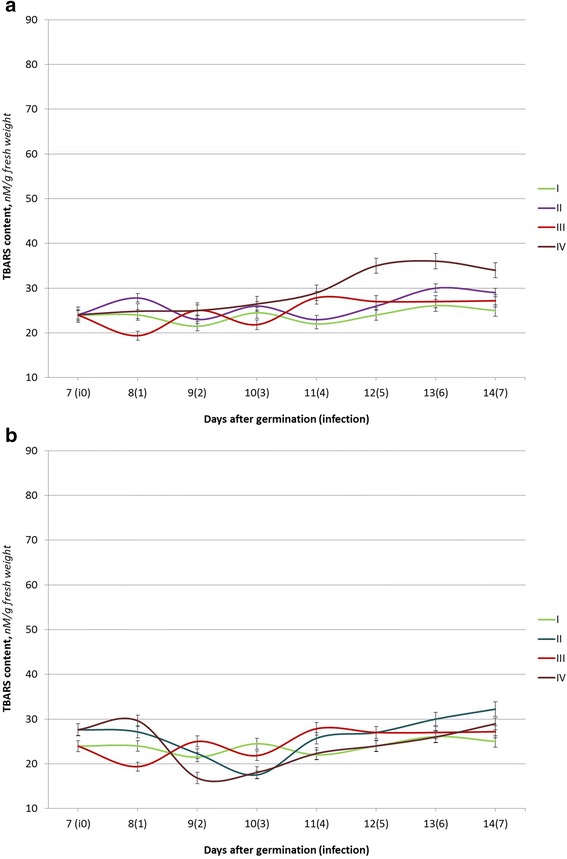
The TBARS content of seedlings of winter wheat (the Roazon variety) under the action of Ag (**a**) or Cu (**b**) nanoparticle solutions and *P. herpotrichoides* infection: *I* control plants (w/o infection); *II* seed treatments with Ag (**a**) or Cu (**b**) nanoparticle solutions; *III P. herpotrichoides* infections; *IV P. herpotrichoides* infections + treatments with Ag (**a**) or Cu (**b**) nanoparticle solutions

**Fig. 3:**
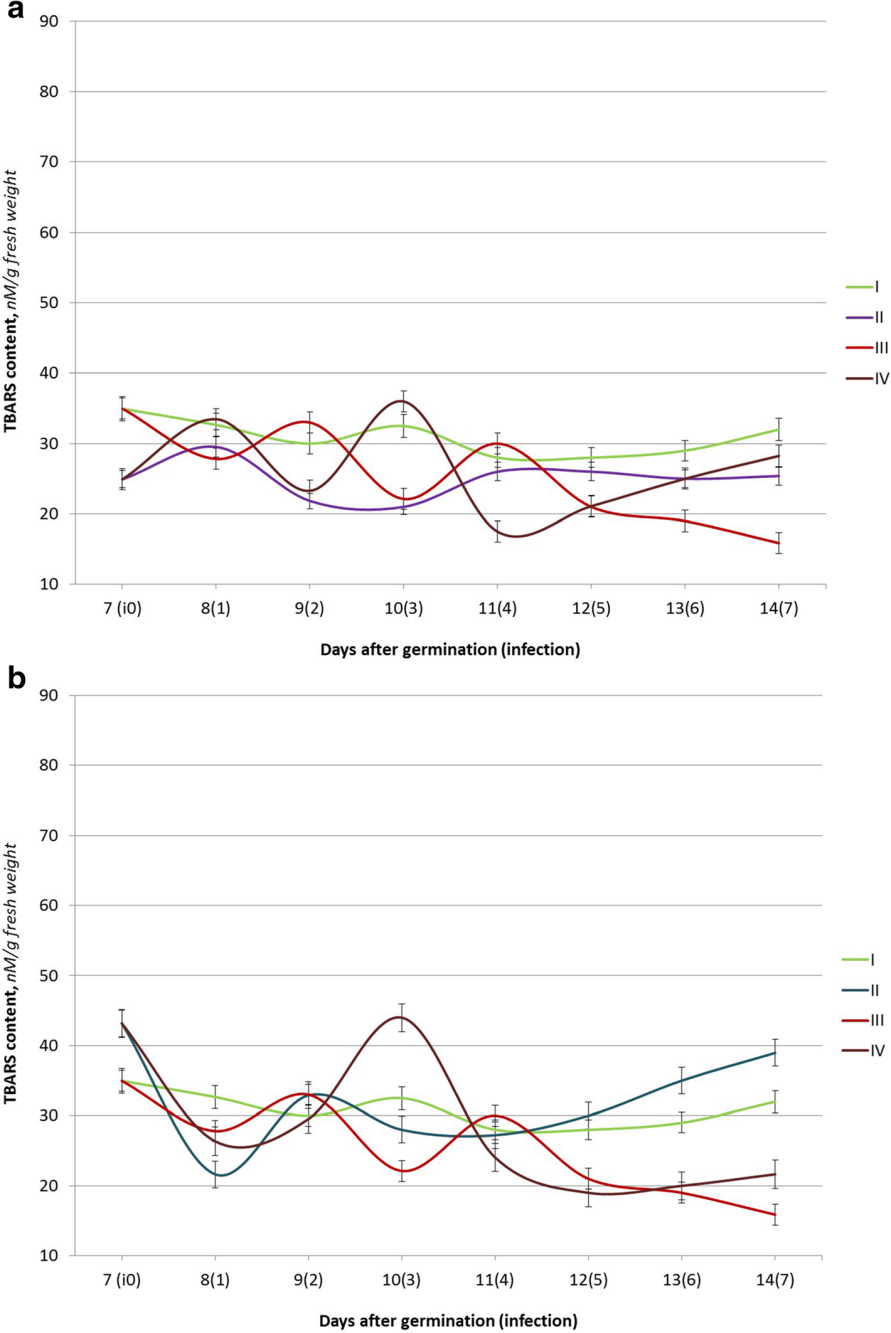
The TBARS content of seedlings of winter wheat (the Renan variety) under the action of Ag (**a**) or Cu (**b**) nanoparticle solutions and *P. herpotrichoides* infection: *I* control plants (w/o infection); *II* seed treatments with Ag (**a**) or Cu (**b**) nanoparticle solutions; *III P. herpotrichoides* infection; *IV P. herpotrichoides* infection + treatments with Ag (**a**) or Cu (**b**) nanoparticle solutions

Seed germination is followed by the activation of metabolic processes and is affected by various environmental factors which lead to the generation of ROS, which are neutralized by the elements of a highly efficient pro/antioxidant system [18]. The relative stabilization of the TBARS content in winter wheat seedlings in uninfected (control) variants was registered on the 9th day after germination for the Myronivska 808 variety (Fig. 1a, b) and on the 8th day after germination for the Roazon (Fig. 2a, b) and the Renan (Fig. 3a, b) varieties.

A significant difference was observed in absolute values: 42.3–50.0 nM/g fresh weight for winter wheat seedlings of the Myronivska 808 variety; 28.0–32.7 nM/g fresh weight for winter wheat seedlings of the Renan variety, and 21.5–26.1 nM/g fresh weight for winter wheat seedlings of the Roazon variety (average value ranges). The data obtained corresponded to the available information regarding the resistance grades of the selected varieties. Thus, the highest level of lipid peroxidation products was observed for the variety highly sensitive to the action of the pathogen, indicating a lower efficiency of its pro/antioxidant system.

A significant increase of the TBARS content was noticed in seedlings of the Myronivska 808 variety, sensitive to this pathogen action, on the day following the inoculation with the pathogenic fungi. The maximum TBARS content (79.5% of the control values) in this variant was registered on the 9th day of the experiment, the second day after infection. This was followed by a gradual reduction of lipid peroxidation products (up to the 81.3% compared to the control on the 6th day).

The infected seedlings of Roazon (relatively resistant) and Renan (relatively intermediate resistance) varieties have a different response to the infection. Thus, two peaks and two lows of TBARS content were observed for both varieties at the 2nd and 4th days after infection and on the 1st and 3rd days, correspondingly.

Furthermore, the peak TBARS content values for the Roazon variety reached were 16.4% and 26.6% and only 10.0 and 7.1% for the Renan variety. The minimum TBARS content values were lower than in the control at 19.3 and 10.8% for the Roazon variety and at 14.9 and 31.9%, respectively, for the Renan variety. In addition, the levels of the TBARS content in the infected seedlings of the relatively resistant Roazon variety were close to the control values during the last 3 days of the experiment, while the TBARS content in the seedlings of the Renan variety had gradually declined and was two times lower than in the control on the 7th day after infection (on the 14th day of the experiment). Overall, the data obtained demonstrates that *P. herpotrichoides* infection causes the lowest fluctuations in the TBARS content in seedlings of relatively resistant varieties.

Pre-sowing seed treatments with colloidal solutions of silver and copper biogenic metal nanoparticles have caused changes in the TBARS content of winter wheat seedlings in all varieties studied, both infected and the controls (uninfected). Thus, a seed treatment with Ag and Cu nanoparticles has triggered similar but slightly earlier changes in the TBARS content in seedlings of the Myronivska 808 variety (both infected and the controls (uninfected) variants) to those observed in the variant with the *P. herpotrichoides* infection but without the use of nanoparticles.

The highest values of the TBARS levels in winter wheat seedlings were observed on the day following infection (the 8th day of experiment) for variants treated with Ag/Cu nanoparticles and the use of Cu nanoparticles combined an infection and in 2 days after infection (the 9th day of experiment) for a variant with Ag nanoparticles and infected.

The last variant (a combination of the Ag nanoparticle seed treatment and seedling infection with *P. herpotrichoides*) had the largest fluctuations in the levels in the dynamics of its lipid peroxidation products (and its growth relatively to the control): the maximum value was 189.4% compared to the control (the 2nd day after inoculation), while the minimum value, 50.2%, was observed on the 4th day after inoculation.

It is worthwhile to note that under the seed treatment with copper nanoparticles, the TBARS content in winter wheat seedlings was higher as compared to the control in infected and uninfected variants and has decreased to the level of the controls only on the 7th day after infection, in variants of seed treatment with Cu nanoparticles and seedlings infected with *P. herpotrichoides*. The data presented designate the state of the variety, sensitivity to the action of *P. herpotrichoides*, as extremely stressful under the studied conditions (influence of the Ag nanoparticles, the Cu nanoparticles, a combination of seedlings infected with *P. herpotrichoides*, and treatments with Ag or Cu nanoparticles). This can be explained by the extremely unstable ROS synthesis/deactivation system in seedlings of different varieties, sensitive to pathogen action, and possible insufficient levels of antioxidant enzymes.

A pre-sowing seed treatment of the relatively resistant variety Roazon with a colloidal solution of silver and copper biogenic metal nanoparticles has resulted in fewer changes in the levels of TBARS content. Thus, under the action of Ag nanoparticles, the fluctuations in the TBARS content were identical to the control ones, with absolute values laying within the margin of error or being slightly higher than the control values (from 6.0 to 16.0%). With seed treatment with Ag nanoparticles and subsequent infection with *P. herpotrichoides*, the TBARS content has increased gradually, exceeding the control values up to 38.08% (with the maximum values being observed on the 13th day of the experiment). With the use of Cu nanoparticles for seed treatments, the TBARS content remained stable both in infected and uninfected seedlings of the Roazon variety as compared to the controls, with the exception of the values observed on the following day (123.4% when compared to the control) and in 2 days (78.3% when compared to the control) after infection in the variant with an impact on the joint stressors.

Winter wheat seedlings of the Renan variety showed a reduction in the content of lipid peroxidation products as compared to the controls (a minimum of 64.6% on the 10th day of the experiment) in variants with pre-sowing seed treatments with Ag nanoparticles. As it was shown by Taran et al. [20], these changes could be related to an increased activity level of the antioxidant enzyme—superoxide dismutase. In seed treatments with Ag nanoparticles and seedlings infected with *P. herpotrichoides*, the fluctuations in the TBARS content was more obvious: the highest value was 110.8% as compared to the control (on the 10th day of the experiment), and the lowest value was 62.5% as compared to the control (on the 11th day of the experiment). Copper nanoparticles had also significantly affected the state of the pro/antioxidant system of the winter wheat seedlings of the Renan variety. The changes in the TBARS content in the variants with seed treatment using Cu nanoparticles with and without infection were similar to the ones observed separately for seedlings exposure to the pathogen (like in the variant with Myronivska 808—a variety, sensitive to pathogen action). The lowest values compared to the control were recorded in the variant with Cu nanoparticles without infection, 66.3% to the control (the 8th day of the experiment), and with infection, 67.9% to the control (the 12th day of the experiment). The highest growth of the TBARS content, 135.4% as compared to the control, was noted on the 3rd day after infection (the 10th day of the experiment) in seedlings pre-treated with nanoparticles followed by the infection.

Surprisingly, the TBARS content in seedlings of winter wheat of the Renan variety on the 13th and 14th days of the experiment was different from the ones of the Myronivska 808 and Roazon varieties and retained more significant differences to the control values. Probably, a prolonged study would clarify the accumulation patterns of lipid peroxidation products as a result of functioning of the pro/antioxidant system.

In order to find out the sole impact of silver and copper nanoparticles on the growth of *P. herpotrichoides* fungi and to understand whether nanoparticles inhibit or activate fungal growth in the plant-pathogen system studied, the next experiment was conducted.

The growth of fungi on an agar nutrient media is characterized by different criteria: the colony diameter (cm), a daily gain in the column diameter (mm/day), a growth coefficient (mm/day), and the radial growth rate (mm/day), which are considered to be more permanent signs when compared to the color and texture of colonies and are, therefore, important criteria for selecting strains [9].

**Fig. 4:**
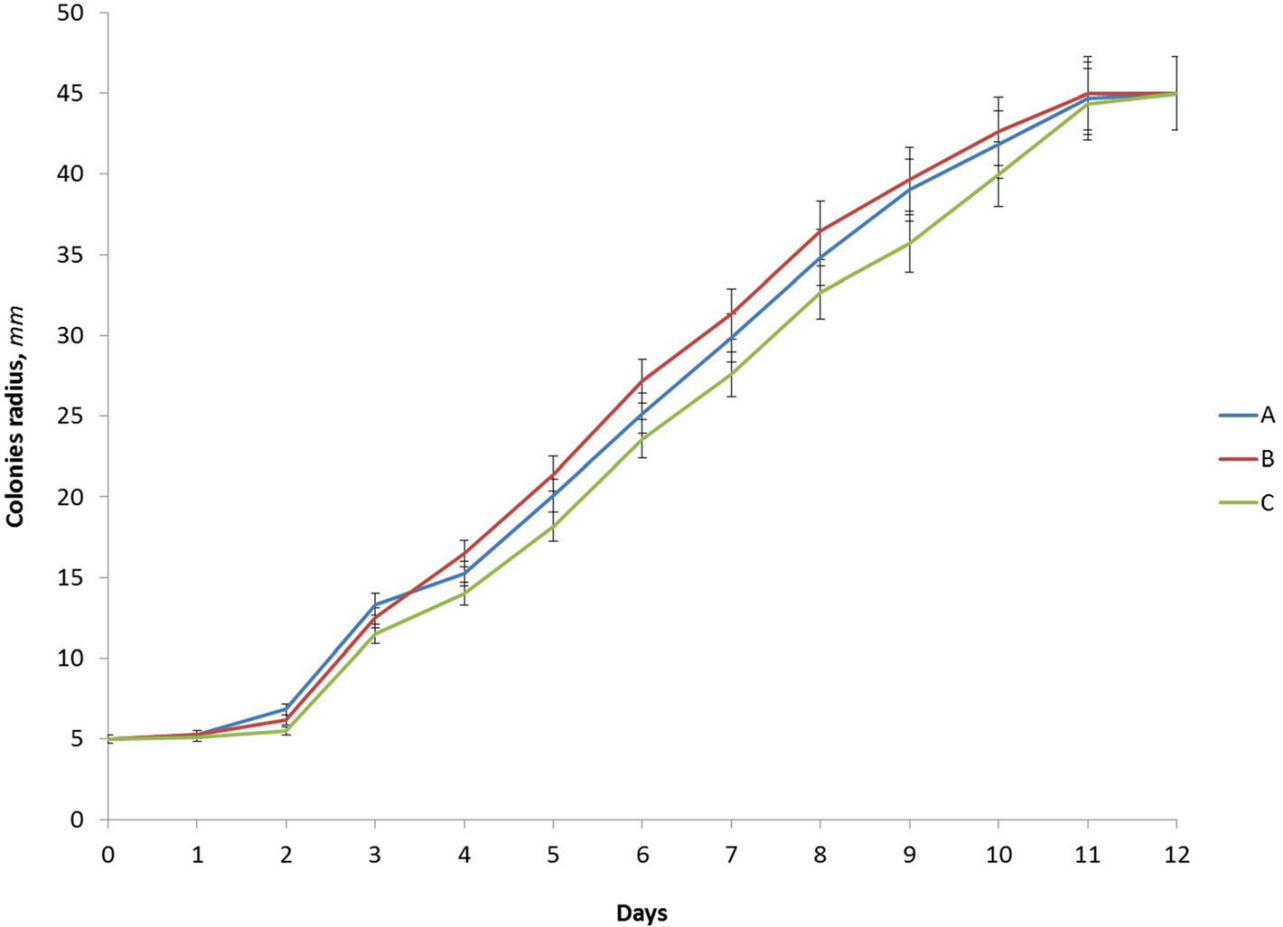
The dynamics of changes in the radius of *P. herpotrichoides* colonies, grown on PDA with the addition of silver or copper nanoparticles: *A* control (without nanoparticles); *B* PDA media with Ag nanoparticles; *C* PDA media with Cu nanoparticles

The dynamics of the changes in radius growth in *P. herpotrichoides*, grown on PDA with the addition of silver or copper nanoparticles, are shown in Fig. 4. The intensification of mycelium growth in all variants was observed on the 3rd day of cultivation, reaching its maximum on the 12th day of cultivation (all variants had reached 45 mm in colony radius); the *P. herpotrichoides* mycelium had fully covered the surface of the Petri dishes. This indicates the absence of a direct impact of silver and copper nanoparticles on the growth of *P. herpotrichoides* mycelium, based on changes in the colony radius.

As for the linear growth of colonies, the average growth rate for *P. herpotrichoides* cultivated on PDA with nanoparticles was the same in both experimental variants: 3.88 ± 0.01 mm/day and 3.88 ± 0.03 mm/day, correspondingly for the Ag and Cu nanoparticles, which was slightly higher than the control without the addition of nanoparticles (3.78 ± 0.11 mm/day) (see the Table 1). The data shown have confirmed our previous conclusions and indicate that silver and copper nanoparticles have no impact on the growth rate of *P. herpotrichoides* mycelium.

**Table 1:** The linear growth rate of *P. herpotrichoides* mycelium on PDA with the addition of silver and copper metal nanoparticles

Variants	The average linear growth rate (*V*_*r*_ ± *m*), mm/day
PDA medium, without nanoparticles (the control)	3.78 ± 0.11
PDA medium, with Ag nanoparticles	3.88 ± 0.01
PDA medium, with Cu nanoparticles	3.88 ± 0.03

**Fig. 5:**
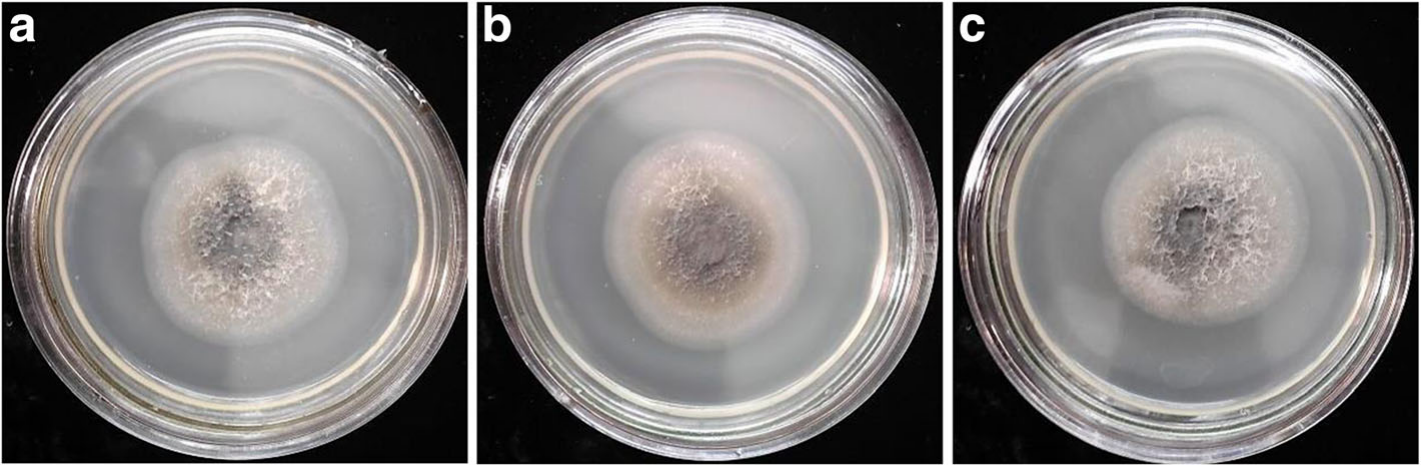
*P. herpotrichoides* colonies, grown on PDA (a 7th day culture) with the addition of silver or copper nanoparticles: **a** control (without nanoparticles); **b** PDA media with Ag nanoparticles; **c** PDA media with Cu nanoparticles

Visual observations of the cultural-morphological characteristics of *P. herpotrichoides* mycelium colonies have revealed no differences between the variants. Thus, all colonies were hairy, with black and brown centers and white halos, dense, and having relatively clear edges (Fig. 5).

Data consolidation indicates that silver and copper nanoparticles previously not investigated in experiments with *P. herpotrichoides* in the proposed concentrations have not affected the growth and development of *P. herpotrichoides* mycelium, namely the colony radius range, the average radial growth rates, and the cultural-morphological characteristics of the *P. herpotrichoides* mycelium. It is likely that higher concentrations of silver and copper nanoparticles may impact growth of *P. herpotrichoides* differently. The results obtained are consistent with the findings of other researchers. In particular, the study by Pirog with coauthors [14] has shown that preparations based on gold, silver, cerium, and zirconium dioxide nanoparticles in concentrations of 0.5–7.5 mg/l had no antimicrobial effect on micromycetes (*Aspergillus niger* P-3, *Fusarium culmorum* T-7, and *Penicillium chrysogenum* F-7) and yeast *Candida scottii* CB-2. Besides, the bactericide action of metal nanoparticles depends on their size as the reduction of nanoparticles size from 10 μm to 10 nm increases the surface area by 10^9^ times, thus allowing one to reduce the concentration of nanoparticles by hundreds of times and thus, simultaneously, increase their antimicrobial properties [8].

## Conclusions

As it was shown, the pre-sowing seed treatments with nonionic colloidal solutions of biogenic metal nanoparticles of Ag and Cu as well as the complex influence of nanoparticles and seedling infections exert a stress load on plants, displaying a dynamic pattern of the changes in the TBARS content similar to the one activated at pathogenesis, but to a different extent. Thus, the variety sensitive to pathogenic action has a significant (100%) increase in TBARS contents, while the range in changes of the TBARS content in seedlings of other varieties was lesser (40%) as compared to the control. It is noteworthy that the impact of nanoparticles and infections in the seedlings of the resistant variety, Roazon, on the TBARS content, was relatively smaller than compared to the other varieties. The maximum intensity of lipid peroxidation was observed in variants with seed treatments with Ag nanoparticles and infection winter wheat seedlings of the Myronivska 808 variety and in seeds treated with Cu nanoparticles and an infection of winter wheat seedlings of the Renan variety. Silver and copper nanoparticles in selected concentrations have not affected the growth and development of *P. herpotrichoides* mycelium. Our findings suggest that the effect of silver and copper nanoparticles (at concentrations recommended by the manufacturer) in the initial stages of the pathological process within wheat-*P. herpotrichoides* interactions is determined by plant responses to the pathogen, but not the phytotoxic action of the studied nanoparticles.
